# The Role of Cytokines in the Functional Activity of Phagocytes in Blood and Colostrum of Diabetic Mothers

**DOI:** 10.1155/2013/590190

**Published:** 2013-12-17

**Authors:** Danny Laura Gomes Fagundes, Eduardo Luzía França, Glilciane Morceli, Marilza Vieira Cunha Rudge, Iracema de Mattos Paranhos Calderon, Adenilda Cristina Honorio-França

**Affiliations:** ^1^Graduate Program in Gynecology, Obstetrics and Mastology of Botucatu Medical School, São Paulo State University (UNESP), Av. Prof. Montenegro Bairro: Distrito de Rubião Junior, s/n, 18618-970 Botucatu, SP, Brazil; ^2^Institute of Biological and Health Science, Federal University of Mato Grosso (UFMT), Rodovia BR070, Km 5 s/n, 78600-000 Barra do Garças, MT, Brazil

## Abstract

Immune response changes induced by diabetes are a risk factor for infections during pregnancy and may modify the development of the newborn's immune system. The present study analyzed colostrum and maternal and cord blood of diabetic women to determine (1) the levels of the cytokines IFN-**γ** and TGF-**β** and (2) phagocytic activity after incubation with cytokines. *Methods.* Colostrum and maternal and cord blood samples were classified into normoglycemic (*N* = 20) and diabetic (*N* = 19) groups. Cytokine levels, superoxide release, rate of phagocytosis, bactericidal activity, and intracellular Ca^2+^ release by phagocytes were analyzed in the samples. Irrespective of glycemic status, IFN-**γ** and TGF-**β** levels were not changed in colostrum and maternal and cord blood. In maternal blood and colostrum, superoxide release by cytokine-stimulated phagocytes was similar between the groups. Compared to spontaneous release, superoxide release was stimulated by IFN-**γ** and TGF-**β** in normoglycemic and diabetic groups. In the diabetic group, cord blood phagocytes incubated with IFN-**γ** exhibited higher phagocytic activity in response to EPEC, and maternal blood exhibited lower microbicidal activity. These data suggest that diabetes interferes in maternal immunological parameters and that IFN-**γ** and TGF-**β** modulate the functional activity of phagocytes in the colostrum, maternal blood, and cord blood of pregnant diabetic women.

## 1. Introduction

Maternal interaction with the fetus is bidirectional. Fetal and placental tissues require suitable environment, under homeostasis, whereas the maternal body is affected by factors related to metabolic adjustments. In this relationship, the fetus receives passive immunity from the mother, which is crucial for newborn adaptation to the extrauterine environment because it provides protection against infectious agents during the first months of life [1, 2].

Cells with phagocytic and microbicidal activity are among the multiple immune components of blood and human milk that play an important role in child protection [3, 4]. A number of studies report that diabetic patients have low phagocytic and microbicidal activity and reactive oxygen species production due to changes in their antioxidant systems. Moreover, the reduction in phagocytic and microbicidal activity of leukocytes is likely related to an increase in blood glucose levels [5–7].

In diabetic individuals, the balance between proinflammatory and anti-inflammatory cytokines is not fully understood. Some studies show that they prioritize the production of proinflammatory cytokines [8], whereas others relate that the production of both cytokine types is increased by diabetes [9].

The cytokine gamma interferon (IFN-*γ*) and transforming growth factor *β* (TGF-*β*) seem to act in the early stages of pregnancy [10, 11] and participate in cellular functions. It is believed that IFN-*γ* promotes the inflammatory reaction that enables trophoblast implantation, whereas TGF-*β* acts on maternal immunological response, embryo implantation, and placental and fetal development [10, 12].

IFN-*γ* and TGF-*β* play a role in the activation and regulation of immune cells. IFN-*γ* promotes the microbicidal response of phagocytes, increasing the expression of surface receptors and rates of phagocytosis [13]. TGF-*β*, in turn, likely induces and maintains the immune tolerance to the fetus [12] and the production of regulatory cells [14]. Nevertheless, the action of these cytokines on the microbicidal activity of blood and colostrum phagocytes of diabetic mothers has yet to be investigated.

The present study analyzed the colostrum and maternal and cord blood of diabetic women in order to determine (1) the levels of the cytokines IFN-*γ* and TGF-*β* and (2) phagocytic activity after incubation with cytokines.

## 2. Materials and Methods

The functional activity of colostrum and maternal and cord blood phagocytes in diabetic women was evaluated in a cross-sectional study. The subjects attended the Diabetes and Pregnancy Facility, School of Medicine Obstetrics Course, UNESP, Botucatu, SP, Brazil. This study was approved by the institutional Research Ethics Committee, and all the subjects gave written informed consent before entering the experimental protocol.

### 2.1. Subjects

Blood and colostrum samples from pregnant women (18–45 years old) were analyzed by maternal glycemic status. According to the results of the 75 g oral glucose tolerance test (OGTT 75 g) [15] and glucose profile (GP) test [16], 39 pregnant women were classified into the following groups: normoglycemic group (normal 75 g OGTT and normal GP; *n* = 20) and diabetes mellitus group (altered GTT 75 g, prior to or during the pregnancy and abnormal GP; *n* = 19). The subjects continued attending the facility, irrespective of diagnosis, and the diabetic patients followed a specific treatment for glycemic control [16].

### 2.2. Blood Sampling and Separation of Blood Cells

Samples of 8 mL of maternal blood were collected prior to the beginning of labor and cord blood at birth in tubes with anticoagulant. We centrifuged them at 160 G for 15 min to separate plasma from the cells. Cells were separated by a Ficoll-Paque gradient (Pharmacia, Uppsala, Sweden), producing preparations with 95% of pure mononuclear cells, analyzed by light microscopy. Purified macrophages were resuspended independently in serum-free medium 199 at a final concentration of 2 × 10^6^ cells/mL. The cells were used immediately for assays of superoxide release, phagocytosis, microbicidal activity, and calcium release. The plasma was stored at −80°C for later glucose and cytokines analysis.

### 2.3. Colostrum Sampling and Separation of Colostral Cells

About 8 mL of colostrum from each woman was collected in sterile plastic tubes between 48 and 72 hours postpartum. The samples were centrifuged (160 G, 4°C) for 10 min, which separated colostrum into three different phases: cell pellet, an intermediate aqueous phase, and a lipid-containing supernatant. The upper fat layer was discarded and the aqueous supernatant was stored at −80°C for later analyses. Cells were separated by a Ficoll-Paque gradient (Pharmacia, Uppsala, Sweden), producing preparations with 98% of pure mononuclear cells, analyzed by light microscopy. Purified macrophages were resuspended independently in serum-free medium 199 at a final concentration of 2 × 10^6^ cells/mL. The cells were used for assays of superoxide release, phagocytosis, microbicidal activity, and calcium release. The colostrum supernatant was stored at −80°C for later glucose and cytokines analysis.

### 2.4. Glucose Determination

Glucose levels were determined by the enzymatic system. Samples of 20 *μ*L colostrum/maternal or cord blood, standard of 100 mg/dL (Doles), were placed in 2.0 mL phosphate buffer solution (0.05 M, pH7.45, with aminoantipyrine 0.03 mM, 15 mM sodium p-hydroxybenzoate, 12 kU/L glucose oxidase, and 0.8 kU/L peroxidase). The suspensions were mixed and incubated for 5 min at 37°C. The reactions were read on a spectrophotometer at 510 nm.

### 2.5. Cytokine Dosage By ELISA (Enzyme-Linked Immunosorbent Assay)

IFN-*γ* concentrations in the colostrum and milk supernatants were determined by an ELISA kit from BioLegend Legend Max (San Diego, USA), and TGF-*β* concentrations were analyzed using an ELISA kit from Enzo Life Sciences (UK). The reaction rates were measured by absorbance in a spectrophotometer with a 450 nm filter. The results were calculated using the standard curve and shown in pg/dL.

### 2.6. *Escherichia Coli* Strain

The enteropathogenic *Escherichia coli* (EPEC) used was isolated from stools of an infant with acute diarrhea (serotype 0111: H^−^ *AL* ^−^, *eae* ^+^, *eaf* ^+^, *bfp* ^+^). This material was prepared and adjusted to 10^7^ bacteria/mL, as previously described by Honorio-França [17].

### 2.7. Treatment of Blood and Colostral Phagocytes with Cytokines

To assess the effect of cytokines (IFN-*γ* and TGF-*β*) on superoxide anion release, phagocytic, microbicidal activity, and intracellular Ca^2+^ release, MN phagocytes (2 × 10^6^ cells/mL) were incubated with 5 *μ*L of cytokines (Sigma St. Louis, USA, final concentration 100 ng/mL) for 1 h at 37°C. The phagocytes were then washed once with 199 medium at 4°C and immediately used in the assays. A control was performed with only 199 medium.

### 2.8. Release of Superoxide Anion

Superoxide release was determined by cytochrome C (Sigma, St. Louis, USA) reduction [17, 18]. Briefly, mononuclear phagocytes (blood and colostrum) and bacteria were mixed and incubated for 30 min for phagocytosis. Cells were then resuspended in PBS containing 2.6 mM CaCl_2_, 2 mM MgCl_2_, and cytochrome C (Sigma, St. Louis, USA; 2 mg/mL). The suspensions (100 *μ*L) were incubated for 60 min at 37°C on culture plates. The reaction rates were measured by absorbance at 550 nm and the results were expressed as nmol/O_2_^−^. All the experiments were performed in duplicate.

### 2.9. Bactericidal Assay

Phagocytosis and microbicidal activity were evaluated by the acridine orange method [19]. Equal volumes of bacteria and cell suspensions were mixed and incubated at 37°C for 30 min under continuous shaking. Phagocytosis was stopped by incubation in ice. To eliminate extracellular bacteria, the suspensions were centrifuged twice (160 ×g, 10 min, 4°C). Cells were resuspended in serum-free 199 medium and centrifuged. The supernatant was discarded and the sediment was dyed with 200 *μ*L of acridine orange (Sigma, St. Louis, USA; 14.4 g/L) for 1 min. The sediment was resuspended in cold 199 medium, washed twice, and observed under immunofluorescence microscope at 400x and 1000x magnification.

The phagocytosis index was calculated by counting the number of cells ingesting at least 3 bacteria in a pool of 100 cells. To determine the bactericidal index, we stained the slides with acridine orange and counted 100 cells with phagocytized bacteria. The bactericidal index is calculated as the ratio between orange-stained (dead) and green-stained (alive) bacteria x100 [4]. All the experiments were performed in duplicate.

### 2.10. Intracellular Ca^2+^ Release Determined by Fluorescence and Flow Cytometry

We performed fluorescence staining at the FACS Calibur (BD, San Jose, USA) to assess intracellular Ca^2+^ release in phagocytes [20]. Cells were loaded with the fluorescent radiometric calcium indicator Fluo3-acetoxymethyl (Fluo3-AM-Sigma, St. Louis, USA). Cell suspensions, pretreated or not with 50 *μ*L of cytokines (Sigma, final concentration of 100 ng/mL), were mixed and incubated at 37°C for 30 min under continuous stirring. Suspensions were centrifuged twice (160 ×g, 10 min, 4°C) and resuspended in PBS containing BSA (5 mg/mL). This suspension was incubated with 5 *μ*L of Fluo-3 (1 *μ*g/mL) for 30 min at 37°C. After incubation, cells were washed twice in PBS containing BSA (5 mg/mL; 160 ×g, 10 min, 4°C) and then analyzed by flow cytometry (FACS Calibur system, BD, San Jose, USA). Calibration and sensitivity were routinely checked using CaliBRITE 3 Beads (BD, Cat. no 340486, USA). Fluo-3 was detected at 530/30 nm filter for intracellular Ca^2+^. The rate of intracellular Ca^2+^ release was expressed in geometric mean fluorescence intensity of Fluo-3. Data shown in the figures correspond to one of several trials performed.

### 2.11. Statistical Analysis

Data were expressed as the mean ± standard deviation (SD). The statistically significant difference was evaluated using the analysis of variance (ANOVA) for superoxide release anion, phagocytosis, bactericidal index, and intracellular Ca^2+^ release in the presence or absence of cytokines and the difference was compared at the statistical significance was considered for a *P*-value less than 0.05.

## 3. Results

Glucose levels in colostrum, maternal blood, and cord blood were higher in hyper- than in normoglycemic women. However, leukocyte retrieval and viability and IFN-*γ* and TGF-*β* levels in maternal blood, cord blood, and colostrum samples were similar between the groups (Table 1).

Diabetic and normoglycemic groups had similar spontaneous superoxide release by mononuclear (MN) phagocytes in colostrum. When exposed to EPEC and cytokines, the phagocytes of both groups increased superoxide release (*P* < 0.05). Irrespective of the presence of cytokines, phagocytes in maternal blood showed the highest superoxide release when exposed to bacteria. In the diabetic group, cord blood phagocytes displayed higher spontaneous superoxide release than those in the normoglycemic group. In the normoglycemic group, cord blood phagocytes exhibited the highest superoxide release when exposed to bacteria and cytokines. In the diabetic group, cytokines did not affect superoxide release by cord blood phagocytes (Table 2).

**Figure 1:**
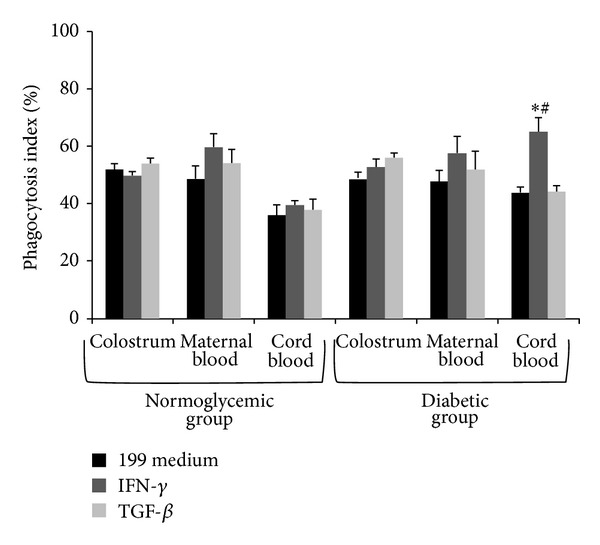
Bacterial phagocytosis by colostrum and maternal and cord blood phagocytes (mean ± SD, *N* = 10 in each treatment and sample), determined by the acridine orange method. Phagocytes were incubated with enteropathogenic *Escherichia coli* (EPEC) in the presence of gamma interferon (IFN-*γ*) and transforming growth factor *β* (TGF-*β*). *indicates differences from the 199 medium and cytokines use within each sample and group; ^#^ indicates differences between normoglycemic and diabetic groups within each treatment (cytokines or 199 medium) and sample (colostrum, maternal blood, or cord blood).

MN phagocytes from colostrum and maternal blood, treated or not with cytokines, exhibited similar phagocytic activity against EPEC, irrespective of glycemic status (Figure 1). Cord blood phagocytes from the diabetic group displayed a higher phagocytic index when exposed to bacteria and IFN-*γ* (*P* < 0.05—Figure 1).

**Figure 2:**
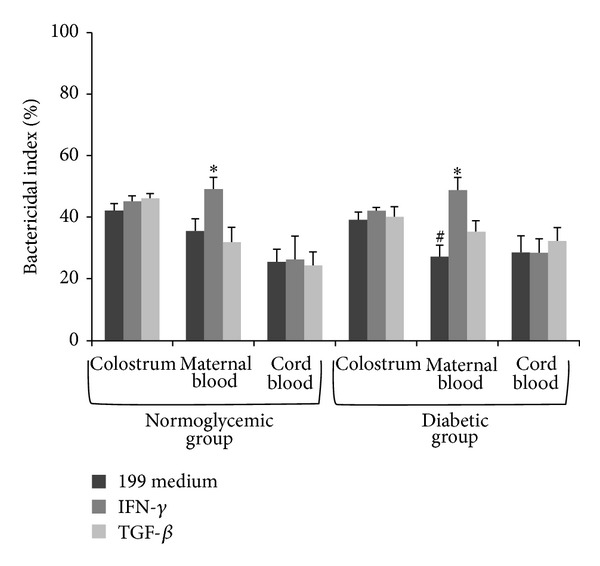
Bactericidal index (mean ± SD, *N* = 10 in each treatment and sample), determined by the acridine orange method. Phagocytes were incubated with enteropathogenic *Escherichia coli* (EPEC) in the presence of gamma interferon (IFN-*γ*) and transforming growth factor *β* (TGF-*β*). *Indicates differences from the 199 medium and cytokines use within each sample and group; ^#^ indicates differences between normoglycemic and diabetic groups within each treatment (cytokines or 199 medium) and sample (colostrum, maternal blood, or cord blood).

In general, maternal blood phagocytes in the diabetic group showed low bactericidal activity against EPEC. Both groups exhibited equivalent rates of EPEC elimination by mononuclear phagocytes when treated with cytokines. Maternal blood phagocytes, in both the groups studied, treated with IFN-*γ* exhibited a higher bactericidal index. The bactericidal index of colostrum and cord blood phagocytes did not vary between the groups (Figure 2).

**Figure 3:**
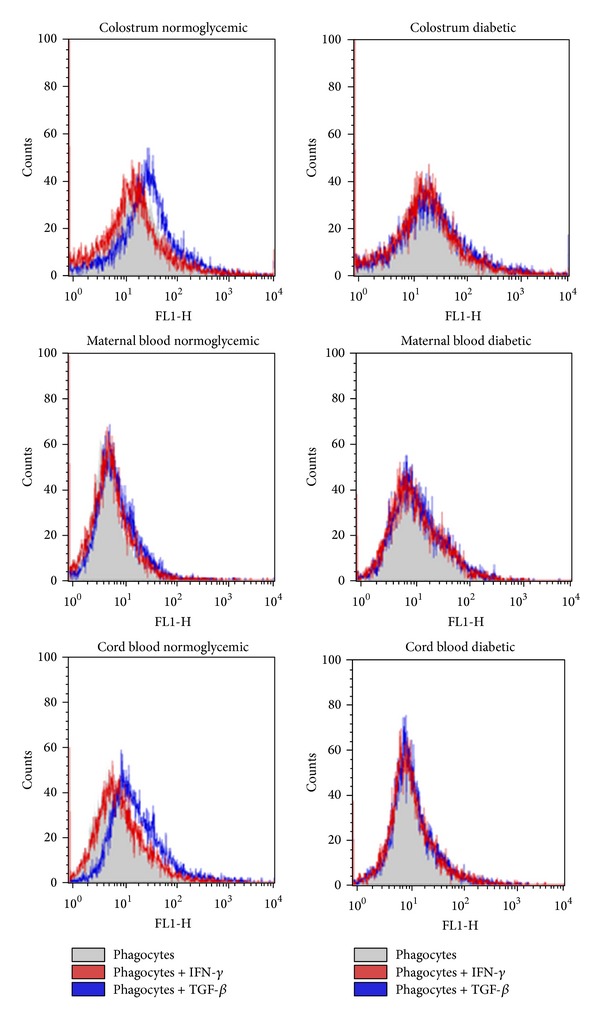
Intracellular Ca^2+^ release by colostral, maternal blood and cord blood phagocytes of diabetic mothers stimulated or not with cytokines. Cells were stained with Fluo-3, and immunofluorescence analyses were carried out by flow cytometry (FACScalibur, Becton Dickinson, USA).

In the diabetic group, maternal blood phagocytes had low intracellular Ca^2+^ release, irrespective of cytokine treatment. In the normoglycemic group, cord blood phagocytes showed lower intracellular Ca^2+^ release than those in maternal blood. In the presence of TGF-*β*, intracellular Ca^2+^ release was higher in cord cells from the normoglycemic group. In the diabetic group, colostrum phagocytes not incubated with cytokines showed lower intracellular Ca^2+^ release when compared with cells from normoglycemic group (Table 3 and Figure 3).

## 4. Discussion

The present study describes IFN-*γ* and TGF-*β* levels in colostrum and maternal and cord blood of diabetic women and how these cytokines affect phagocytic activity in the maternal body and the gestation products evaluated.

Several factors affect cytokine production and action during pregnancy [21]. One of these cytokines, IFN-*γ*, is implicated in the network of mediators of diabetes [22]. In the present study, however, hyperglycemia did not affect the levels of IFN-*γ* and TGF-*β* in colostrum and maternal and cord blood.

A number of studies show that part of the clinical picture of diabetic patients is associated with excessive release of proinflammatory cytokines. Cytokines exert profound effects on the biological signaling and regulation of important physiological processes that are compromised by diabetes [22]. Cytokines may also be related to phagocyte activation and production of reactive oxygen species [23].

In the present study, the cytokines tested modulated superoxide release. Colostrum phagocytes and maternal blood phagocytes increased superoxide release in both groups studied. On the other hand, in the diabetic group, cord blood phagocytes exhibited the highest spontaneous superoxide release, suggesting phagocyte activation irrespective of cytokine presence. A number of mechanisms possibly contribute to the formation of these reactive oxygen-free radicals. Glucose oxidation is believed to be the main source of free radicals [24]. Hyperglycemia also promotes lipid peroxidation by a superoxide-dependent pathway, producing free radicals [25, 26]. Another important source of free radicals in diabetic individuals is the products of glucose-protein interaction [27].

The functional activity of phagocytes was assessed in the colostrum and blood samples of diabetic patients [1, 3, 7, 28] and animals with induced diabetes [29, 30]. Phagocytes play an important role in host defense. Here, we showed that, in the presence of cytokines, phagocytes from colostrum and maternal blood exhibit similar phagocytic activity against EPEC, irrespective of the women's glycemic status. In the diabetic group, cord blood phagocytes increase phagocytosis rate in the presence of IFN-*γ*.

Phagocytosis and microbicidal activity of phagocytes in colostrum and blood, with production of active oxygen metabolites such as free radicals, consist of an important defense mechanism against a number of bacterial [4, 31], fungal [32], and protozoal infections [33]. In the present study, we found that phagocytes in the blood of diabetic mothers exhibited low bactericidal activity against EPEC. However, after incubation with cytokines, bacterial elimination by MN phagocytes of diabetic women increased to rates similar to those obtained in the normoglycemic group.

Earlier studies have reported that diabetic patients have low phagocytic and microbicidal activity due to derangements in prooxidative systems. An increase in blood glucose levels is indeed related to a decrease in the phagocytic activity of leukocytes [5–7]. On the other hand, cytokines such as IFN-*γ* act primarily on macrophages by activating their phagocytic and microbicidal abilities [13, 34]. The present study is the first to report the effects of IFN-*γ* and TGF-*β* on the functional activity of colostrum and maternal and cord blood phagocytes of diabetic mothers.

Interestingly, cord blood phagocytes exposed to EPEC and stimulated by IFN-*γ* showed an increase in phagocytosis but not in their bactericidal activity. A number of studies report that these cells display low bactericidal activity because they lack nonspecific surface receptors, while others argue that they are still too immature to exhibit this response [1].

In diabetic patients, cytokine production may be associated with a number of processes such as alterations in intracellular Ca^+2^ by phagocytes, which is triggered by advanced glycation and products [35] and hyperglycemia [36]. In the present study, cytokines did not stimulate intracellular Ca^2+^ release by maternal blood phagocytes in the diabetic group, and in the normoglycemic group, TGF-*β* increased intracellular Ca^2+^ release in cord cells.

Transforming growth factor *β* (TGF-*β*) is involved in the balance of maternal immune response and deployment process and produced early in the maternal-fetal interface by the embryo and decidua [10, 14].

Although colostral phagocytes of diabetic women decrease intracellular Ca^2+^ release in relation to normoglycemic women [7], we show here that colostrum phagocytes, irrespective of cytokine incubation, increase intracellular Ca^2+^ release in relation to maternal and cord blood phagocytes.

The maternal transfer of immune components during pregnancy and breastfeeding represents a remarkable immunologic interaction between the mother and newborn. Breast milk is an excellent source of immunological components, and it decreases the high rates of maternal and infant complications. Adequate maternal glycemic control during pregnancy and breastfeeding duration in diabetic mothers is crucial to ensure that the immunity components act against the infections.

## 5. Conclusion

Our findings support the hypothesis that diabetes interferes in maternal immunological parameters due to alterations in glucose metabolism and that IFN-*γ* and TGF-*β* can modulate the functional activity of colostrum, maternal blood, and cord blood phagocytes of diabetic women and these cytokines can be alternative for use in future clinical applications in diabetic patients.

## Figures and Tables

**Table 1:** Mean (±SD) glucose level, leukocyte count, viability, IFN-*γ*, and TGF-*β* concentrations in colostrum, maternal blood, and cord blood from normoglycemic and diabetic women.

Parameter	Sample	Normoglycemic	Diabetic
Glucose level (mg/dL)	Colostrum	66.0 ± 7.4	114.2 ± 9.6*
	Maternal blood	90.5 ± 8.6	122.1 ± 8.9*
	Cord blood	69.0 ± 7.3	87.0 ± 8.5*

Mononuclear phagocytes count (×10^6^ cell/mL)	Colostrum	2.8 ± 0.5	2.4 ± 0.8
	Maternal blood	4.8 ± 0.7	5.1 ± 0.5
	Cord blood	2.9 ± 0.6	3.2 ± 0.9

Mononuclear phagocytes viability (%)	Colostrum	92.0 ± 3.5	90.0 ± 2.2
	Maternal blood	91.0 ± 2.4	90.0 ± 3.3
	Cord blood	90.0 ± 2.6	91.0 ± 2.7

IFN-*γ* (pg/mL)	Colostrum	9.2 ± 1.5	7.9 ± 3.1
	Maternal blood	8.2 ± 1.7	8.4 ± 0.5
	Cord blood	8.9 ± 1.8	7.5 ± 2.6

TGF-*β* (pg/mL)	Colostrum	22.1 ± 1.6	24.0 ± 4.3
	Maternal blood	26.1 ± 4.7	26.6 ± 3.7
	Cord blood	26.0 ± 4.6	27.6 ± 4.4

*Statistical differences in glucose levels between normoglycemic and diabetic groups, considering the same kind of samples.

**Table 2:** Superoxide release by colostrum and mononuclear phagocytes of blood (mean ± SD, *N* = 10 in each treatment).

Phagocytes	Incubated with	Superoxide release (nmol)	
		Normoglycemic	Diabetic
Colostrum	PBS	1.7 ± 0.4	2.0 ± 0.5
	Bacteria	4.4 ± 0.8*	4.1 ± 0.6*
	Bacteria plus IFN-*γ*	4.6 ± 0.5*	3.5 ± 0.4^∗+^
	Bacteria plus TGF-*β*	4.1 ± 0.5*	3.6 ± 0.1^∗+^

Maternal blood	PBS	1.8 ± 0.2	1.7 ± 0.2
	Bacteria	3.5 ± 0.8*	3.8 ± 0.4*
	Bacteria plus IFN-*γ*	4.1 ± 0.8*	3.7 ± 0.4*
	Bacteria plus TGF-*β*	4.3 ± 0.5*	4.5 ± 0.1^∗#^

Cord blood	PBS	3.2 ± 0.5^#^	4.9 ± 1.1^+#^
	Bacteria	4.8 ± 0.8*	4.6 ± 1.0
	Bacteria plus IFN-*γ*	5.4 ± 0.7*	5.1 ± 0.5^#^
	Bacteria plus TGF-*β*	4.6 ± 0.3*	5.6 ± 0.1^#+^

Colostrum and blood mononuclear cells were treated or not with cytokines, in the presence or absence of EPEC. *Indicates differences between phagocytes treated or not with cytokines and incubated with bacteria and the control (without bacteria) within each group and sample (colostrum, maternal blood, or cord blood); ^#^indicates differences between sample (colostrum, maternal blood, and cord blood) within each treatment (cytokines or PBS) and group; and ^+^indicates intergroup differences within each treatment (cytokines or PBS) and sample (colostrum, maternal blood, or cord blood).

**Table 3:** Intracellular Ca^2+^ release by mononuclear (MN) colostrum phagocytes of diabetic mothers indicated by fluorescence intensity.

Phagocytes	Incubated with	Fluorescence intensity (%)	
		Normoglycemic	Diabetic
Colostrum	PBS	20.2 ± 0.3	17.8 ± 1.5^+^
	IFN-*γ*	22.0 ± 2.4	19.6 ± 5.2
	TGF-*β*	20.4 ± 0.56	18.1 ± 3.28

Maternal blood	PBS	14.2 ± 2.3^#^	9.6 ± 2.3^#+^
	IFN-*γ*	16.6 ± 2.6^#^	10.2 ± 2.2^#+^
	TGF-*β*	15.5 ± 3.8^#^	9.1 ± 2.6^#+^

Cord blood	PBS	10.2 ± 2.1^#^	10.8 ± 1.4^#^
	IFN-*γ*	10.3 ± 2.5^#^	10.9 ± 1.4^#^
	TGF-*β*	19.6 ± 5.2*	10.3 ± 1.6^#+^

Colostrum and blood mononuclear cells were preincubated or not with cytokines. *Indicates differences between phagocytes incubated with cytokines and the control (PBS) within each group and sample, ^#^indicates differences between samples (colostrum, maternal blood, and cord blood) within each treatment (cytokines or PBS) and group; and ^+^indicates differences between normo- and diabetic groups within each treatment (cytokines or PBS) and sample (colostrum, maternal blood, or cord blood).
